# Techniques for early diagnosis of oral squamous cell carcinoma: Systematic review

**DOI:** 10.4317/medoral.20347

**Published:** 2015-02-07

**Authors:** Clàudia Carreras-Torras, Cosme Gay-Escoda

**Affiliations:** 1 DDS Dentistry License , School of Dentistry, University of Barcelona (Spain); 2 MD,DDS,MS, PhD, EBOS. Chairman and Professor of Oral and Maxillofacial Surgery, School of Dentistry, University of Barcelona. Director of Master’s Degree Program in Oral Surgery and Implantology (EFHRE International University/FUCSO). Coordinator/Researcher of the IDIBELL Institute. Head of Oral and Maxillofacial Surgery and Implantology Department of the Teknon Medical Center, Barcelona (Spain)

## Abstract

**Background and objectives:**

The diagnosis of early oral potentially malignant disorders (OPMD) and oral squamous cell carcinoma (OSCC) is of paramount clinical importance given the mortality rate of late stage disease. The aim of this study is to review the literature to assess the current situation and progress in this area.

**Material and Methods:**

A search in Cochrane and PubMed (January 2006 to December 2013) has been used with the key words “*squamous cell carcinoma”, “early diagnosis” “oral cavity”, “Potentially Malignant Disorders” y “premalignant lesions*”. The inclusion criteria were the use of techniques for early diagnosis of OSCC and OPMD, 7 years aged articles and publications written in English, French or Spanish. The exclusion criteria were case reports and studies in other languages.

**Results:**

Out of the 89 studies obtained initially from the search 60 articles were selected to be included in the systematic review: 1 metaanalysis, 17 systematic reviews, 35 prospective studies, 5 retrospective studies, 1 consensus and 1 semi-structured interviews.

**Conclusions:**

The best diagnostic technique is that which we have sufficient experience and training. Definitely tissue biopsy and histopathological examination should remain the gold standard for oral cancer diagnose. In this systematic review it has not been found sufficient scientific evidence on the majority of proposed techniques for early diagnosis of OSCC, therefore more extensive and exhaustive studies are needed.

**Key words:** Squamous cell carcinoma, early diagnosis, oral cavity, potentially malignant disorders, premalignant lesions.

## Introduction

Oral cavity cancer (OC) is a major health concern the world over (1), is defined as a malignant neoplasm on the lip or in the mouth (2) that ranks from the sixth to eight most common cancer around the world (3), with 300.000 new cases reported every year (4-5). The 5-year survival rate has not improved for these patients remains at <50%, early diagnosis and treatment of malignancies usually optimizes long-term cure and survival (6). When the tumour disease is beyond and advanced stage (stage III-IV), the prognosis falls to 30-50%; whereas for diseases discovered early (stage I) the survival rate is 80% (5). Tissues that may be involved as the site of origin include the labial and buccal mucosa, the anterior two-thirds of the tongue, the retromolar pad, the floor of the mouth, the gingiva and the palate (7). The most common form of OC is Oral Squamous Cell Carcinoma (OSCC) (8), witch accounts for over 90% of malignant lesions in the mouth (3,9-12). The 5-years survival rate for OSCC has remained at approximately 50% over the past three decades (11,13-15).

The previous situations to the occurrence of a cancer are called pre malignant lesions. Rethman *et al*. (7) defines them like a morphologically altered tissue noted on clinical examination in which cancer is more likely to occur than in normal tissue; such lesions could be pre cancerous or pre malignant and may exhibit epithelial dysplasia (ED) on histopathologic examination. The World Health Organization (WHO) favors the term “Oral Potentially Malignant Disorders” (OPMD) for clinically recognized disease in which oral cancer may arise (9) as proposed Warnakurasuriya and cols. (16). This classification include: oral leukoplakia, oral erythroplakia, oral lichen planus, nicotine stomatitis, tobacco pouch keratosis and oral submucous fibrosis (2). Leukoplakia is the most common OPMD (17-19) and its worldwide prevalence is approximately 2,6% (20).

According to literature data, OPMD might turn into carcinoma in a percentage varying between 5-18% of cases (4,21). The presence of moderate or severe dysplasia has been accepted to have the greatest likelihood for malignant transformation (14,19), ranging from 11-36% with a mean time of 33,6 months (14). As early cancer frequently been reported to be asymptomatic, the presence of symptoms that are not strongly indicative of carcinoma might induce general practitioners to interpret the symptoms incorrectly and consequently fail to refer the patients for investigation, resulting in diagnostic delay (22).

The main risk factors, exogenous and endogenous, which are involved in the transformation of dysplastic oral epithelium are tobacco (1,7,15,23) which may play a synergistic role in oral tumorigenesis (23,24) and it’s associated with 75% of all cases of OC (12). Alcohol, has also been implicated in oral carcinogenesis not like a etiological specific factor but like adjuvant, acting both independently as well as synergically with smoking (7,23), being the risk of developing OC 30 times higher when associated of tobacco and alcohol (21). It has also been observed increased risk of OC people who have certain inherited diseases, such as Fanconi anemia (7) or leukoplakia patients (25). Human papilloma virus has more recently been identified as a leading etiologic risk factor in oropharyngeal SCC (15,24). Other viruses such as epstein barr virus or hepatitis C may also be related to the OC (12).

The diagnosis of OPMD and OSCC is of paramount importance given the mortality rate of late stage disease (26). Early recognition and diagnosis of OSCC might improve patient survival and reduce treatment-related morbidity (27). Therefore it is vital to know which methods are currently applied and his diagnostic accuracy.

In our experience based on the literature we have to emphasize the importance of methods toluidine blue staining and lugol staining for the screening of pre malignant and malignant lesions of the oral cavity. The objective of this study is to review the literature to assess the current situation and progress in the early diagnosis of OSCC and OPMD.

## Material and Methods

In this systematic review, a search in Cochrane and MEDLINE (PubMed) databases (January 2006 to December 2013) has been used with the key words “squamous cell carcinoma”, “*early diagnosis” “oral cavity”, “Potentially Malignant Disorders” y “premalignant lesions*”.

The inclusion criteria were the use of techniques for early diagnosis of OSCC or OPMD, 7 years aged articles and publications written in English, French or Spanish. The exclusion criteria were case reports and studies in other languages. The articles selection was agreed by consensus between the two authors; first by reading of titles and abstracts of the found bibliographic cites to identify the most relevant studies and then, by means of reading the full-text article. A summary that synthesizes the techniques for early diagnosis of OSCC and TOPM that we have identified has been made ( Table 1).

**Table 1:**
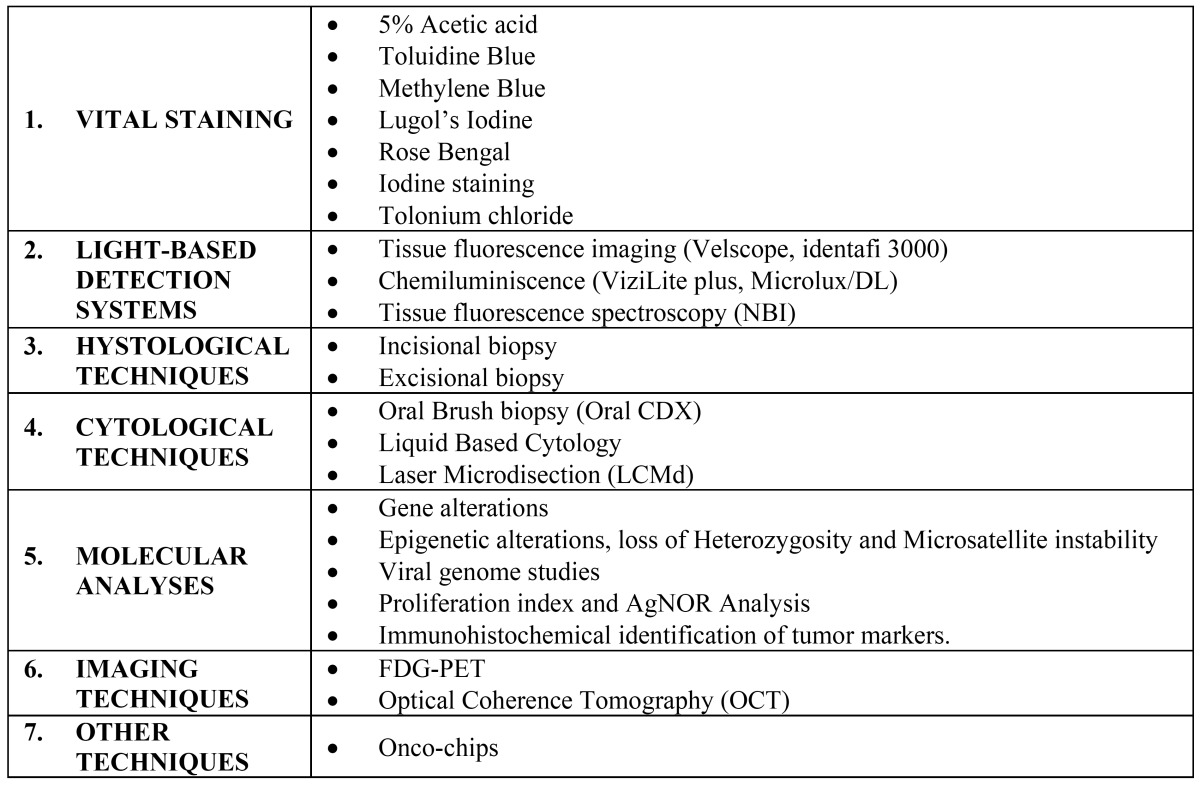
Techniques that contribute to the diagnosis of oral cancer in addition to coe.

## Results

Out of the 89 studies obtained initially from the search (12 with Cochrane and 77 with Medline), 29 of these 89 articles were excluded due to the lack of data and/or lack of direct relationship with the subject and finally, 60 articles were selected to be included in the systematic review: 1 metaanalysis, 17 systematic reviews, 35 prospective studies, 5 retrospective studies, 1 consensus and 1 semi-structured interviews. The biography also cite some important articles referenced in the 60 selected articles.

To establish a correct evaluation of any diagnostic test, several parameters must be considered: The sensitivity of a test, is the proportion of people who test positive for a specific disease among a group of people who have the disease. Specificity is the proportion of people who test negative for a specific disease among a group of people who do not have the disease. False positive is an erroneously positive test or screening result. False negative is an erroneously negative test or screening result. Positive predictive value (PPV) is the proportion of people in a specified population with positive test results who have the disease. Negative predictive value (NPV) is the proportion of people in a specified population with negative test results who are disease-free.

The current protocol for detecting OPMD by conventional oral examination (COE) involves visual inspection of the oral cavity and tactile examination of head and neck lymph nodes (11). The criteria for suspicion of an OPMD or OSCC include changes in surface texture, loss of surface integrity, color, size, contour deviation or mobility of intraoral or extra oral structures (28). A recent meta-analysis (29) reported 93% of sensitivity for the EOC, but specificity was only 31%. Therefore, EOC cannot reliably differentiate between benign and dysplastic lesions, and this is probably due to the fact that a number of benign conditions mimic oral malignancies.

The gold standard for COC or TOPM diagnosis remains incisional biopsy (single or multiple) of the suspicious tissue and his histopathological examination (4,8,13,20,28,30). However, screening by taking random biopsies of both clinically normal and suspect oral tissue is unpractical, since this causes serious discomfort to the patient and is not suitable for repeated sampling at multiple sites (30). As in other fields of medicine, diagnostic techniques of the oral cavity are going towards not painful, noninvasive, simple methods, inexpensive and accesive methods (31).

## Discussion

In order to facilitate the understanding of this section, we have divided according to the most relevant techniques or methods of early diagnosis of OSCC and OPMD that we have found in this systematic review.

1.-Vital Staining

Are techniques that use a range of pigments with a tendency to focus on cells with high reproductive rate, as neoplastic cells, indicating the most suitable areas to practice biopsies and be controlled and examined.

Bhalang *et al*. (10) conducted a study to assess the diagnostic accuracy of 5% acetic acid for COCE diagnosis. The sensitivity and specificity were 83,33% and 84,21% respectively. Sankaranarayanan *et al*. (32) who investigated the detection of cervical cancer using 4% acetic acid reported a sensitivity and specificity of 88% and 78% respectively. Although the use of this vital stain for the screening of CB appear to be interesting, are needed more studies to support its use (10).

Vital tissue staining with toluidine blue is the most used method for early detection of OPMD and OSCC. Is a cationic meth achromatic dye that may selectively blind to free anionic groups such as sulphate, phosphate and carboxyl ate radicals of large molecules. It can be useful due to the binding ability to the phosphate groups of the nucleic acids to be retained in the intercellular spaces of dysplastic epithelium (15). Epstein *et al*. (33), explain that toluidine blue sensitivity and specificity had a 92,5% and 63,2% respectively. One metaanalysis of Rosenberg *et al*. (34) previously published reported sensitivity ranged from 93,5% to 97,8% and the specificity ranged from 73,3% to 92,9%. It’s practical, rapid, inexpensive, and effective adjunct diagnostic tool in mucosal disease. Its showed use in high-risk patients examined by experienced providers reduced the number of biopsies of benign lesions by approximately 50% and identified all severe dysplasia and OSCC lesions (15). The clinical application of toluidine blue has been shown to be selective staining of premalignant and malignant lesions (28), has a high sensitivity, but a low specificity due to the false positive that generates (29). So, is recommended as an adjunct to the clinical examination of oral mucosal lesions, specifically in high-risk patients by expert providers, but when stain is retained, all suspicious lesions should undergo biopsy (15). Moreover, that was shown to have toxicity to fibroblasts (13).

Another kind of dye material, methylene blue, dyes cells with acidophilic characteristic and may penetrate into cells with abnormal increase in nucleic acid, thus resulting in different uptake between normal and highly dysplastic/malignant cells. Chen *et al*. (13) investigated his diagnostic accuracy and results revealed sensitivity of 90%, specificity of 69%, PPV of 74% and NPV of 87%. The 90% sensitivity obtained was no less than the 72-100% sensitivities reported whit toluidine blue staining in other studies. The difference and also the most significant problem was the fact that there where three false negatives (10%). Authors conclude that considering its low toxicity and the fact that it is cheaper than toluidine blue it may be convenient to substitute toluidine blue in large-scale oral screening in high-risk patients. However, the histopathological report by incisional biopsy must remain the gold standard. Remembering that the histopathological study of the entire lesion with margins and depth control will give us the diagnosis of certainty.

Lugol’s Iodine staining having affinity for glycogen epithelial cells, cells that contain more glycogen are going to retain more stain than those with lower content, such as carcinoma cells, in which the reaction with Lugol’s it’s not going to produce or very dim. In one study of Epstein *et al*. (33) Lugol’s iodine was proposed as CB screening technique and was described a sensitivity of 87% and a specificity of 84%. Another study (35) observed sensitivity, specificity, PPV and NPV for identifying TOPM with Lugol’s iodine of 34,5%, 100%, 60,7% and 100% respectively. In some studies it is highlighted that the combined use of Lugol’s iodine and toluidine blue increases the specificity; Peng *et al*. (35) evaluated the results of double staining with Lugol’s Iodine and Methylene Blue values and they obtained 97,7%, 100%, 97,8% and 100% respectively. In this study, this double staining significantly improves detection of OSCC and TOPM because combinations of both stains weakens the defects of each staining separately.

Du *et al*. (36) studied the efficacy of Rose Bengal staining. It was observed that the sensitivity for detect COCE and ED with the Rose Bengal stain was significantly higher that with visual examination (93,9% vs. 72,7%) while specificity was similar with both methods (73,7% vs. 76,8%). In this study there were false positive cases that were mostly caused by inflammation or trauma, fact that is observed in a similar way with the toluidine blue stain, although it seems more promising in this study rose bengal staining to toluidine blue to detect the TOPM. They conclude that Rose Bengal staining is a valuable diagnostic test in detection of TOPM and OC. However further studies in larger samples sizes are needed.

Watanabe *et al*. (37) verified the effectiveness of Iodine staining and obtained a sensitivity and specificity for detecting OSCC of 100% and 59,6%, respectively. The authors explain that iodine staining can be easily performed and that helps in decision of surgical margin for locally resection. They conclude that the use of iodine staining as a part of clinical examination may be beneficial for early detection of COCE in high-risk patients.

Finally, Patton *et al*. (38) in their study refer to the use of tolonium chloride that may bind preferentially to tissues undergoing rapid cell division. Rosenberg *et al*. (34) reported an overall sensitivity of 93,5% and specificity of 73,3%.

2.-Light-based detection systems 

They are devices featuring special light sources designed according to principles of tissue reflectance and tissue auto fluorescence to enhance the oral examination process (7), they function under the assumption that mucosal tissues undergoing abnormal metabolic or structural changes have different absorbance and reflectance profiles when exposed to various forms of light or energy (39).

2.1.-Techniques of auto fluorescence 

The technique involves illumination of suspicious lesions with monochromatic light and recording the fluorescence spectra emitted by endogenous tissue fluorophores (1). The presence of cellular alterations will change the concentrations of fluorophores, which will affect the scattering and absorption of light in the tissue, thus resulting in changes in color that can be observed visually (39). Exposure to blue light spectra may maximize a differential profile in areas undergoing neoplastic change in which a loss of fluorescence visualization is reported (40).

Jayanthi *et al*. (1) evaluates the potential of a multivariate statistical algorithm to classify oral mucosa from auto fluorescence spectral features recorded in vivo. They explore the potential of linear discriminant analysis, to predict group membership of a sample with unknown group. Marked differences were seen between auto fluorescence spectra of healthy tissues (500nm) and malignant lesions (635-705nm). This method was able to differentiate pre-malignant ED from OSCC, benign hyperplasia from ED and hyperplasia from normal with overall sensitivities of 86%, 78% and 92%, and specificities of 90%, 100% and 100% respectively. The results obtained confirm the advantages of using multivariate statistical analysis on linear discriminant analysis spectral data for noninvasive diagnosis of OPMD.

VELscope® is a device for direct visualization of changes in tissue fluorescence in the oral cavity that produce near ultraviolet/blue light between 400 and 460 nm. Digital image processing images can be used to outline suspicious regions in real time. Rana *et al*. (4), in his study showed that using the VELscope® leads to higher sensitivity (100% vs. 17%), but to a lower specificity (74% vs. 97%) compared with COE. The major lack of the study was the large number of false-positive test results. In one other study of Koch *et al*. (40) show a high sensitivity (97%) and specificity (95,8%) to diagnose OSCC. The PPV was calculated at 41% and NPV at 75-80%. In another study of Moro *et al*. (21) they obtains a sensibility up to 100% and specificity up to 93% to individualization of oral cancer in populations at risk. Finally Lane *et al*. (41) using histology at the gold standard, demonstrated a 98% sensitivity and a 100% specificity for discriminating ED and OC from normal oral mucosa.

VELscope® is a simple, noninvasive examination test of the oral mucosa with the ability to help locate malignant oral lesions and find the right location for a biopsy (4), showing often limits of the lesion wider with the autofluorescence than those shown at clinical examination (21). The device could help to identify any type of pathological oral, but could not reliably distinguish benign oral lesions from dysplasia or OSCC (40), however it is very helpful in the diagnosis of OC (4,20). The results of the device should be interpreted with caution due to the issue of frequently occurring FP results and his low specificity (4), therefore, lesions showing a red auto fluorescence signal should need further clarification via histology (40). The device should not be used in the hands of inexperienced clinicians and cannot be a replacement for the gold standard of any histological evaluation (4,19).

McNamara *et al*. (42) concluded in his study that the results suggest that COE is more valid that auto fluorescence examination (with VELscope®) in routine screening for OPMD. They do not support use of this device in routine screening assessment of asymptomatic dental patients. And they believe that careful, systematic visual and tactile examination of the entire oral cavity on a regular basis remains the gold standard for early detection of OPMD.

Another auto fluorescence device is Identafi 3000® that combines anatomical imaging with fluorescence, fiber optics and con focal microscopy to map and delineate precisely the lesion in the area being screened. The advantage of this device over the VELscope® is its small size and easy accessibility to all tissues in the oral cavity. One study of Schwarz *et al*. (43) demonstrated a sensitivity of 82% and a specificity of 87% in differentiating between neoplastic and non-neoplastic oral conditions. Another study of McGee *et al*. (44) showed that healthy tissue could be discriminated from ED and OC with 100% sensitivity and 91,4% specificity. Finally Roblyer *et al*. (45) reported a capacity for discriminating between normal oral mucosa and ED or OSCC of 96 to 100% sensitivity and 91 to 96 specificity. Further investigations of the device are needed to evaluate the clinical utility of this device (11).

2.2.-Autofluorescence techniques combined with endoscopic visualization methods

Narrow band imaging is an endoscopic visualization technology using the lighting created with optical interference filters with different spectral ranges of low frequency. This technology utilize the concept that the wavelength of light determines the depth of penetration, therefore, the reflected photons constituting the images coming from different depths (surface and deeper layers) of the object that is explored. Bhatia *et al*. (11) did a literature research to evaluate the use of this technology in the oral cavity. He found only a few papers that have evaluated the use of narrow band imaging, and reported a Sensitivity, specificity, PPV, NPV for detecting OC ranged from 95 to 96%, 97 to 100%, 91 to 100% and 93 to 99% respectively. In comparison the ranges for COE with white light were generally lower at 51 to 64%, 96 to 100%, 82 to 100% and 87 to 90% respectively. Nguyen *et al*. (46) conducted a prospective study to evaluate combined auto fluorescence and narrow band imaging for detection of OPMD and OC, they found a sensitivity for detecting moderate ED or worse at 96% with, which was better than white light which had only 38%.

2.3.-Chemiluminiscence 

Among the tissue-reflectance-based devices we found: The ViziLite Plus® that use a disposable chemiluminescent light packet. The *Microlux/DL® and Orascoptic DK®* that use a reusable, battery-powered light-emitting diode (LED) light source that provides a similar blue-white (440 nm range) illumination. Under the blue-white illumination, abnormal squamous epithelium is reported to be distinctly white (acetowhite). Vizilite Plus® also provides a toluidine blue solution which is intended to mark an acetowhite lesion for subsequent biopsy (7,39).

It seems that the chemiluminescent exam using ViziLite Plus® helped to enhance the brightness, sharpness, texture and size of serious pathology lesions in comparison with COE under incandescent light (7,28,38,47). Some studies concluded that examination with the ViziLite did not change the diagnosis (4) and that they have not been shown to enhance the practitioner’s ability to identify OPMD not visible under normal operatory lighting (7). Different studies (7,35,39,47) show that ViziLite® device have high sensitivity (at 100%) but low specificity (0 to 14%) and PPV (18 to 80%) when researchers confirmed its findings through histopathological examination. The sensitivity and specificity data for chemiluminiscent examination is not useful because only patients with visible lesions were included in the different studies, for the moment, the utility of enhanced visual findings in low-risk populations is not known.

That’s conclude Lingen *et al*. (39) the evidence that supports the use of reflective fluorescence systems to aid the detection of OPMD is currently quite sparse. Well-controled clinical trials are needed that specifically investigate the ability of those devices to detect OPMD that are invisible by COE alone.

3.- Histological Techniques 

As already said, the best diagnostic method for OPMD or OSCC lesions is incisional (IB) or excisional biopsy (EB). One of the most useful prognostic indicators of malignancy is the severity of epithelial dysplasia; this is assessed with a biopsy, which allows histological examination and categorization according to features defined by the World Health Organization (WHO), classically into mild, moderate and severe. Histopathology also helps clinicians decide whether or not to make a split from the injuries, according to the perceived risk of malignant transformation. It also identifies the OSCC, even when lesions are not visible clinically. IB may not provide a representative sample of tissue from which the degree of dysplasia or presence of OSCC can be assessed, so rates of malignant transformation might be more accurately measured from EB specimens. In this study we will focus on the new techniques of tissue procurement, focusing on the laser.

Goodson et Thomson (48) assessed the correlation between diagnosis of dysplasia from IB and EB specimen. There was a significant correlation between the results of diagnostic incisional, and laser excision, biopsy specimens, but 15 patients (9%), without high-risk features, had signs of occult invasive carcinoma in the excision specimens. Time interval between IB and laser excision in this study was 6 weeks, which suggest that focus of OSCC were present but missed at IB, presumably because of a sampling error. They explain that in 54% of cases histological examination of IB specimens was accurate in predicting what they found on laser excision, however, in 28% of cases dysplasia was more severe in the laser excised specimen than in the IB specimen. In this study it’s not possible the presence of intraobserver variability by pathologists because the same team of specialist oral pathologists graded both incisional and excisional specimens, thus the differences reflect the inadequacy of incional biopsy specimens for the diagnosis of oral pre cancerous lesions. They conclude that complete excision of the lesion remains essential, not only to establish diagnosis but also to facilitate early efficacious treatment of both, dysplastic and early neoplastic lesions, particularly at a stage when OSCC is clinically undetectable.

It is important to remember that when making a BE must be verified that the margins and the depth of tissue resected is disease-free, so if the BE is made with cold scalpel 1mm safety-margin should be respected, but if we made with CO2 laser, safety margins should be extended even to the 5mm to allow the pathologist be certain that excision was complete (49).

4.-Cytological Techniques

Cytopathology is the microscopic study of cell samples collected from mucosal surfaces (via smears, scrapings or lavage) or from internal sites via fine-needle aspiration (39). The different articles reviewed, basically talks about two techniques, oral exfoliative cytology, which is the study of cells that flake off (naturally or artificially) from the oral mucosa, and liquid based cytology, where the sampling instrument is introduced into a liquid medium immediately being fixed and avoiding sample degeneration over the air.

The OralCDx® Brush Test system uses a specialized brush that collects transepithelial cellular samples composed of free cells and clusters. These samples are fixed, stained and analyzed microscopically by a pathologist with a computer-based imaging system help (7,8,38), allowing to evaluate lesions that do not immediately raise suspicion of OC (7). Rethman *et al*. (7), explains that Brush Test may help the practitioner identify the presence of atypical cells in seemingly innocuous mucosal lesions, but alerts of the high number of FP results, frequently obtained when this test is performed on inflammatory or reactive lesions. Fontes *et al*. (50) evaluated the utility of oral cytopathology in the diagnosis of OSCC and earned a 83,1% of sensitivity, 100% of specificity, 100% of PPV and 49% of NPV.

Kämmerer *et al*. (51) using Cytobrush®Plus GT obtain a 55% of sensitivity and a specificity of 100% in comparison with histology. The PPV and NPV were 100% and 80% respectively. Koch *et al*. (5) also using Cytobrush®Plus GT explain that depending of cytologic criteria of malignancy used the diagnostic accuracy varies. By defining all dysplastic or malignant cytopathologic findings as positive, the sensitivity was increased to 95,2%, at the expense of the specificity, which was reduced from 94,9% to 82,3%. Separately analyzing OSCC of less than 20 mm, the sensitivity was reduced by 88,5% to 78%, and the specificity by 86,4% to 74,5%, as compared when all lesions of all tumor stages were considered.

The cytological study of oral cavity cells is simple and rapid, non-aggressive and relatively painless (52) that appears to be helpful in establishing a more definitive diagnosis in high-risk mucosal lesions (8,38,51). This test it seems of no value in detecting mucosal changes that are not readily visible to the naked eye (8). According to Patton et al. (38) data are insufficient to assess the Brush test’s utility in low-risk population or clinically innocuous lesions. In contrast Kämmerer *et al*. (51) in their study were able to correctly classify all low-risk lesions. There is insufficient evidence to support a recommendation for or against the use of Oral brush test in seemingly innocuous mucosal lesions, In the case of “atypical” result or positive result (ED or OSCC) it should be biopsied immediately for a better diagnosis (7).

Delavarian *et al*. (31) studied the diagnostic accuracy of liquid based citology technique in detection of ED/OSCC using a specialized oral brush (OralCDx® Brush). This technique suggests a higher sensitivity and specificity compared with COE, with witch was in agreement in 88,4% of cases. Brush diagnosis it was in 92,3% with histopathological findings. The results obtained (sensitivity 88,8%, specificity 100%, PPV 100% and NPV 80%) suggest that this technique is a suitable test for clinical use, and that permits eliminate some of disadvantages of the conventional brush. It is vital to understand that cytology has a low NPV, a negative result does not exclude ED or OSCC, repeated biopsy is required if the first biopsy proves negative (9).

5.-Molecular Analyses

Grading of OPMD by molecular methods seems more promising because reduce the inter- and intraobserver variability of histology (30). OC arise as a result of the accumulation of genetic alterations in proto-oncogenes and tumor suppressor genes (24,30). These genetic changes are of value to predict the risk for malignant progression. On the other hand, numerous chromosomal regions have been associated with early OSCC carcinogenesis and should therefore be analyzed in parallel (30). Lingen *et al*. (24) highlight that development of molecularly based approaches to identify predictive biomarkers could be used to improve the potential for early detection, prognostication and intervention of OC.

5.1.-Gene alterations 

Most of the oral cavity carcinogens are mutagenic agents that may cause changes in gene and chromosome structure by point mutations, deletions, insertions and rearrangements. These genetic alterations can be used as targets for detecting tumour cells in clinical samples (52).

Mutations in the tumour suppressor gene p53 are the most frequent genetic alterations in OSCC (10,52,53) with published figures ranging from 35% to 94% (10,53). His function is to regulate a cell cycle checkpoint and the induction of apoptosis in response to DNA damage (10). However, other authors consider that the high number of point mutations, which can be found in p53, limit its potential clinical application (52). Even if its predictive value has been controversial, p53 mutations may be an important event, early or late in the progression of OPMD (53). It has been demonstrated the potential clinical application of oral cytology to detects them (52), cause elevated transcription of the mutant p53 gene contributes to the overall high levels of the mutant protein in tumor cells and results in the accumulation of this protein in the nucleus that can be detected immunohistochemically (20).

Aneuploidy is another genetic alteration that refers to the change in chromosomal number (result from gene dose imbalance, loss of TSG, gain of tumor promoting genes or oncogenes, or formation of fusion genes) (24). Aneuploidy is observed in 20-92% of oral dysplasia (24,54). Donadini *et al*. (54) demonstrated that OPMD that could be clinically classified without ED at histology, contained alredy DNA aneuploidy sublines in 23% of the cases. In the study of Kämmere *et al*. (51) the detection of aneuploidy, which is the basis of DNA-Image cytometry (DNA-ICM), reported 70% sensitivity, 100% specificity, 100% PPV and 86% NPV. The combination of DNA-ICM with Brush Biopsy showed a sensitivity of 76% and a specificity of 100%. The predominant reason for FP results in this study was sampling errors with insufficient cells. In other study (6) the sensitivity of DNA-aneuploidy on oral smears for the detection of cancer cells was 90%, the specificity 100%, PPV 100% and NPV 93% (6). It seems that DNA-ICM has the potential to substantially improve sensitivity, therefore should not be used to rule out malignancy, when lesions are already clinically suspicious for OC but rather as an adjunct to improve the quality of brush biopsy as a screening instrument (51).

Finally, another gene alteration that is related to OC is miRNA. Lingen *et al*. (24) conclude that for the moment there are insufficient evidence available to delinate recommendations regarding the clinical utility of miRNA expression and the prediction of whether a OPMD will progress to OSCC (24).

5.2.-Epigenetic alterations, loss of heterozygosity and microsatellite instability

The applicability of other molecular markers such as epigenetic alterations (hypermethylation of promoter regions) and genomic instability such as loss of hetrozygosity (LOH) and microsatellite instability (MSI) has also been studied.

The main epigenetic modification in tumours is methylation. Rosas *et al*. (55) studied the methylation patterns of p16, MGMT and DAP-K genes in smears of patients suffering from head and neck cancer, using a methylation specific Polymerase Chain Reaction (PCR). They explains that this technique allows sensitive and efficient detection of tumoral DNA. Huang *et al*. (56) that use PCR techniques to amplify DNA from exfoliated cytology samples from oral carcinomas, for analysis of restriction-fragment length polymorphisms. They found that 66% of the tumors studied showed LOH at one position in the p53 sequence. Nunes *et al*. (57) find LOH in 84% of the samples, though with differences depending on tumour stage. Other studies have been used PCR and RFLPs to detect micro satellite markers, i.e. short repetitive DNA sequences, these demonstrated that alterations in certain regions of chromosomes 3p, 9p, 11q, 17p are associated with development of OSCC (52). Bremmer *et al*. (25) in his study detected allelic inestability at these regions using microsatellite markers in exfoliated cell samples of 40% with a 78% of sensitivity and a PPV of 100% (24). Partridge *et al*. (58) observed that LOH at 3p and 9p in 90% of cases progress to cancer.

Bremmer *et al*. (30) applied a novel genetic assay, “the multiplex ligation-dependent probe amplification”, that enables the measurement of gains and losses at 40 different chromosomal location in one PCR reaction using 150 ng DNA. The assay was correlated to loss of heterozigosity analysis using micro satellite markers. They conclude that this technique is a sensitive, reliable, high-throughput and easy-to-perform, enabling the detection of genetic alteration on small noninvasive samples and can be considered a promising method for population-based screening of OPMD in the oral cavity. Lingen *et al*. (24), says that comparison among existent studies is challenged by methodologic differences, adjustment for confounders, and controls, thus, the clinical utility of LOH in 3p and 9p as an effective screen for progression of OPMD to OSCC requires prospective validation.

5.3.-Viral genome studies

A significant proportion of oropharyngeal cancers (40-60%) have human papilloma virus DNA integrated within their genomic DNA. Archival cytology slides can also be used for this DNA detection with “in situ” hybridation. The diagnostic of metastatic lesions usually is determined by fine-needle aspiration. With this technique is used alcohol-fixed, archival, cytopathological material to study the presence of HPV-DNA. It presence was correlated with the origin of metastatic lesions (52).

5.4.-Proliferation index and AgNOR Analysis 

Remmerbach *et al*. (59) demonstrated the validity of oral cytology for analyzing the number of keratinized cells and the nucleolar activity (AgNORs), the authors concluded in this study that AgNOR analysis may be used as a routine method for diagnosing oral cancer. Another study. (6) apply for the first time a multi modal cell analysis, that was based on the sequential application of multiple staining of identical, slide-based cells and repeated relocalization and measurements of their diagnostic features, resulting in multi parametric features of individuals cells. The stepwise application of the two additional approaches (morphology, DNA content, AgNOR) increased the specificity of conventional cytologic diagnosis from 92,6% to 100%. The study demonstrated that this methode may become a sensitive and highly specific, objective, and reproducible adjuvant diagnostic tool for the identification of neoplastic changes in oral smears that contain only a few abnormal cells.

5.5.-Immunohistochemical identification of tumor markers

The identification of tumoral markers, notably cytokeratins in smears from the oral cavity provides useful information on cell differentiation status, but its potential for early diagnosis of oral cancer is limited. However, certain cytokeratins, such as K8 and K19 are useful if not definitive indicators of malignancy, particularly if their presence is interpreted in conjunction with other information, such as DNA profile (52).

For the other hand, Vascular endothelial growth factor (VEGF) plays a central role in regulating angiogenesis in solid tumors and is tightly associated with the angiogenic switch being crucial in the progression of the ED to invasive OSCC. Serum VEGF-A levels have been reported to be elevated in OSCC and have also been correlated with lymph node metastasis and clinical staging. Nayak *et al*. (60) make a inmunohistochimical study to evaluate the expression of circulating VEGF-A (by ELISA assay) and in tissues (using antibodies against VEGF-A and CD-34). Serum VEGF-A levels and immunohistochemical VEGF-A expression showed more than 50-fold increase in OMPD and OSCC in comparison with controls. VEGF-A levels in serum correlated in a linear fashion with the tissue expression in oral pre-malignant and malignant lesions. This seems to be an important finding for predicting progression of OMPD to OSCC. However this would require a well-controlled large scale, multi centric study in different geographical locations along with clinic-epidemiological, etiological factors and follow up studies for serum VEGF expression in post treatment cases.

Finally, also may be important the immunocytochemical of the mini chromosome maintenance (MCM) proteins. They seem to be sensitive and specific biomarkers of cell cycle entry that are essential for eukaryotic DNA replication. Scott et al. (61) conducted a study in which they observed striking differences in expression of this proteins in the surface layers of epithelium showing severe/moderate ED or OSCC, compared to mildly ED or benign lesions. They undertook MCM immunocytochemistry of oral smears and they cytological data were fully consistent with the histopathological observations and indicate that MCM-positive epithelial cells are likely to be present in smears from OSCC but not in scrapes of mild ED and benign keratosis. They conclude that the strong clinical performance and ready interpretation of stained liquid based citology samples make MCMs particularly strong markers for high-throughput screening of high-risk patients.

6.-Imaging diagnostic techniques

The imaging diagnostic techniques are divided into radiographic techniques, nuclear medicine, magnetic resonance and ultrasonography. In this work we review some of nuclear medicine techniques, focusing on: positron emission tomography (PET) that it’s a functional imaging technique that provides information about tissue metabolism and optical coherence tomography (OCT) that is a noninvasive high-resolution imaging modality capable of cross-sectional imaging of biological tissue using back-scattered signals reflected from different layers within the tissue to reconstruct structural images.

Finally we will comment polarimetry a technique that measured the polarization effects of the scattered light from bacterial suspensions to yield useful information to characterize the sample. This technique allows to visualize different useful factors for differentiating between cancerous tissues and his homologous benign.

Liao *et al*. (62) prospectively examined the value of positron emission tomography using the fludeoxyglucose (FDG) molecule immediately before postoperative radiotherapy/concurrent chemo radiotherapy to detect residual/relapsing disease in the early post surgical follow-up period in high-risk OSCC patients. Of the patients who underwent the second scan 24% had unexpected, newly discovered lesions. At two months rates of neck control, distant metastases, and disease free survival were significantly higher in patients who received a second PET scan than in those who did not. The authors conclude that the findings support the clinical value of FDG-PET for defending treatment strategy in OSCC patients with both extra capsular spread and nodal standardized uptake value.

Hamdoon *et al*. (63) in his study conclude that OCT achieve a sensitivity, a specificity, PPV and NPV for detecting OPMD and OC of 85%, 78%, 86,5% and 77,5% respectively. In contrast, Jerjes *et al*. (64) confirms the feasibility of using OCT to identify architectural changes in malignant tissues but reported that its ability to differentiate between different oral mucosal abnormalities was poor. Further research is required on the potential application of OCT to improve and define excisional margins during surgical management of OPMDs and OSCCs (11).

Ahn *et al*. (65), determine a multimodality approach to noninvasive diagnosis of OPMDs and OSCCs in hamsters, “in vivo” polarimetry of the oral mucosa was used to acquire mueller matrix images providing quantitative information on epithelial tissues and OCT was used to map epithelial and sub epithelial changes throughout carcinogenesis demonstrating the feasibility of diagnostic imaging within the oral cavity using these modalities. From OCT images, surface and subsurface structure including blood vessels were clearly visible with epithelial and sub epithelial changes evident in the OCT images paralleling histopathological status. Polarimetry techniques identified a four to five times increased retardance in sites with SCC and two to three times greater retardance in dysplastic sites than in the normal tissues. Taken together, these two techniques could provide useful information for screening patients for oral cancer, and it’s particularly useful for mapping areas of field cancerization with multiple lesions, as well as lesion margins.

7.-Other new Approaches

Finally found one article of Mehrotra et Yadav (23) who talk about the “*Onco-chips*”, which consist of a full array of small cells in which genes suspected to be associated with cancer are introduced. Onco-chips are the new concept consisting of several reliable diagnostic head and neck cancer markers, wich may be used to diagnose cancer. The treatment of cells with therapeutic chemicals, has been shown to produce specific changes in gene expression. It is a technique that is in development at the moment but that is potentially interesting.

## Conclusion

The best diagnostic technique is that which we have sufficient experience and training. Definitely tissue biopsy and histopathological examination should remain the gold standard for oral cancer diagnose.

With oral cytology we can obtain single cells that can be analyzed using sophisticated techniques such as cytomorphometry and molecular analysis or using more simple techniques such as toluidine blue (dye most used) or rose bengal (which has proved more promising), with all these techniques have been achieved very interesting results. On the other hand, optical techniques and diagnostic techniques for imaging have also proved particularly useful, but their results are not yet clinically relevant.

In this systematic review it has not been found sufficient scientific evidence on the majority of proposed techniques for early diagnosis of OPMD and OSCC, therefore more extensive and exhaustive studies are needed.
